# Comparison of Mid-Term Clinical and Radiologic Outcomes Between Measured Resection Technique and Gap Balanced Technique After Total Knee Arthroplasty Using Medial Stabilizing Technique for Severe Varus Knee: A Propensity Score-Matched Analysis

**DOI:** 10.3390/jcm14238450

**Published:** 2025-11-28

**Authors:** Sung-Sahn Lee, Juyong Oh, Young-Wan Moon

**Affiliations:** 1 Department of Orthopaedic Surgery, Ilsan Paik Hospital, Inje University School of Medicine, Goyangsi 10380, Republic of Korea; sungsahnlee@gmail.com; 2 Department of Orthopaedic Surgery, Samsung Medical Center, Sungkyunkwan University School of Medicine, Seoul 06351, Republic of Korea; dhwndyd007@naver.com

**Keywords:** knee joint, arthroplasty, replacement, surgery, treatment outcome

## Abstract

**Background:** The purpose was to compare clinical and radiologic outcomes in patients who underwent total knee arthroplasty (TKA) using the medial stabilizing technique (MST) for severe varus knee with either measured resection technique (MRT) or gap balance technique (GBT). **Methods:** Between February 2018 and August 2020, a total of 236 knees with an HKA angle greater than 15° were enrolled in this study. Propensity score matching (1:1) was done based on age, sex, body mass index (BMI), and preoperative hip-knee-ankle (HKA) angle. 67 knees were enrolled in MRT (group M) and GBT (group G), respectively. The mean follow-up duration was 76.2 and 72 months in Groups M and G. Clinical and radiologic outcomes, including hip-knee-ankle (HKA) angle, joint line distance, and femoral component rotation angle (FCRA), were compared between the groups. **Results:** Preoperative clinical and radiological measurements had shown no statistical significance between both groups. The postoperative radiologic measurements, including HKA angle, joint line distance, and FCRA, were not significantly different. Postoperative WOMAC index, KSKS, and KSFS were also not significantly different between both groups. **Conclusions:** In patients with severe varus deformity undergoing TKA with MST, clinical and radiographic outcomes—including limb alignment, joint line position, and femoral component rotation—were comparable between MRT and GBT on mid-term follow-up data.

## 1. Introduction

Total knee arthroplasty (TKA) remains an effective treatment option for patients with end-stage knee osteoarthritis. The practice of performing TKA has increased globally over the past few decades [1,2]. TKA showed rapid increases of 407% between 2001 and 2010 in South Korea [3]. However, inpatient TKA procedural volume was significantly decreased during the COVID-19 pandemic period [4,5].

In neutrally aligned TKA, the two most commonly employed techniques are the measured resection technique (MRT) and the gap balancing technique (GBT) [6]. Numerous studies have compared these approaches. GBT has been reported to provide improved coronal stability and more accurate femoral component rotational alignment; however, this technique may result in postoperative joint line elevation [7,8]. Despite the conceptual differences between the two techniques, several investigations have demonstrated no significant differences in postoperative knee function or patient satisfaction [6,9].

Severe varus knee deformity with medial compartmental osteoarthritis may arise from a variety of etiologies including constitutional knee varus, extra-articular deformity, and chronic insufficiency of anterior cruciate ligament [10,11]. Conventional TKA in the setting of severe preoperative varus deformity is widely recognized as challenging. One of the primary concerns is the potential need for extensive medial collateral ligament (MCL) release to achieve a balanced mediolateral gap. Excessive release of the MCL has been associated with decreased internal rotation during flexion, mid-flexion instability, and abnormal anterior translation of the femur in deep flexion. Medial instability after TKA has been suggested as a cause of postoperative knee pain and suboptimal functional outcomes [12,13].

The medial stabilizing technique (MST) for TKA was introduced to address these potential issues related to extensive medial release [11,14]. The principle of MST is to minimize medial soft tissue release while permitting controlled lateral laxity. It was demonstrated that a normal knee should be tighter at the medial side and looser at the lateral side [15]. Several studies have reported that MST TKA yields comparable outcomes to conventional TKA, without the disadvantage of joint line elevation [14,16]. Furthermore, MST TKA performed in patients with severe preoperative varus deformity has demonstrated clinical, functional, and radiographic results comparable to those in patients with mild varus. However, limited evidence exists regarding the comparison of postoperative outcomes between MRT and GBT in the context of severe varus deformity treated with MST.

The aim of this study was to compare the clinical and radiographic mid-term outcomes of MRT and GBT in patients with severe varus knees undergoing TKA using the MST. We hypothesized that the two techniques would yield similar clinical and radiologic results, including changes in joint line elevation.

## 2. Materials and Methods

### 2.1. Study Design and Patients

This retrospective comparative study utilized prospectively collected data. Patients who underwent mechanically aligned MRT or GBT TKA with MST between February 2018 and August 2020 were evaluated. The inclusion criteria were: (1) medial compartment osteoarthritis rather than other disease including rheumatoid arthritis, (2) preoperative varus deformity with a hip–knee–ankle (HKA) angle greater than 15° [11,17] and (3) a minimum follow-up of 5 years. Exclusion criteria were: (1) extra-articular deformity due to prior femoral or tibial injury and (2) incomplete clinical or radiographic data.

**Figure 1:**
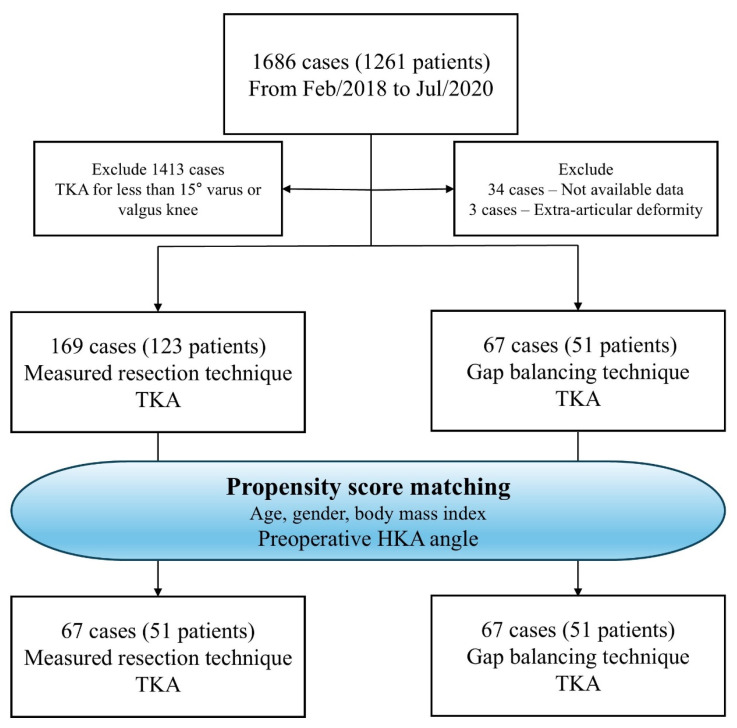
Flow chart describing the patients enrolled in the study.

After applying these criteria, 169 knees (123 patients) were included in the MRT group (Group M), and 67 knees (51 patients) in the GBT group (Group G). Propensity score matching (1:1) was applied based on age, sex, body mass index (BMI), and preoperative HKA angle, resulting in 67 knees in each group (Figure 1). The study was approved by the Institutional Review Board of our institution.

### 2.2. Surgical Technique

All procedures were performed by the senior author (Y-W.M.). A pneumatic tourniquet was applied during whole surgery. A medial parapatellar arthrotomy was employed, followed by meticulous osteophyte removal. The deep medial collateral ligament (MCL) was released to facilitate implantation, but additional medial soft-tissue release was avoided. Physiological lateral laxity was permitted to prevent excessive medial release, consistent with previous reports [5,11]. Distal femoral and proximal tibial cuts were performed perpendicular to the mechanical axes. For MRT TKA, femoral rotation was determined using the surgical transepicondylar axis, posterior condylar axis, and Whiteside’s line. For GBT TKA, anterior and posterior femoral cuts were performed parallel to the proximal tibia. To avoid excessive external rotation, the femoral component was limited to within 3° of the surgical transepicondylar axis. Following bone preparation, a posterior-stabilized (PS) Lospa prosthesis (Corentec Corp., Seoul, Republic of Korea) was implanted in Group M, and a Physica prosthesis (Lima Corp., Udine, Italy) in Group G. Patellar resurfacing was not performed.

All patients followed the same standardized rehabilitation protocol. Isometric quadriceps and active ankle exercises were initiated on the first postoperative day. Active and passive range-of-motion (ROM) exercises began on the second postoperative day.

### 2.3. Clinical and Radiographic Assessments

ROM was assessed using a goniometer. Clinical outcomes included the Western Ontario and McMaster Universities Osteoarthritis Index (WOMAC), the Knee Society Knee Score (KSKS), and the Knee Society Function Score (KSFS), all of which were recorded preoperatively and at the final follow-up visit [12,13,14,15]. Changes from baseline were analyzed within each cohort, and between-group comparisons were performed for KSKS, KSFS, and WOMAC. In addition, any postoperative complications associated with TKA were documented throughout the follow-up period.

**Figure 2:**
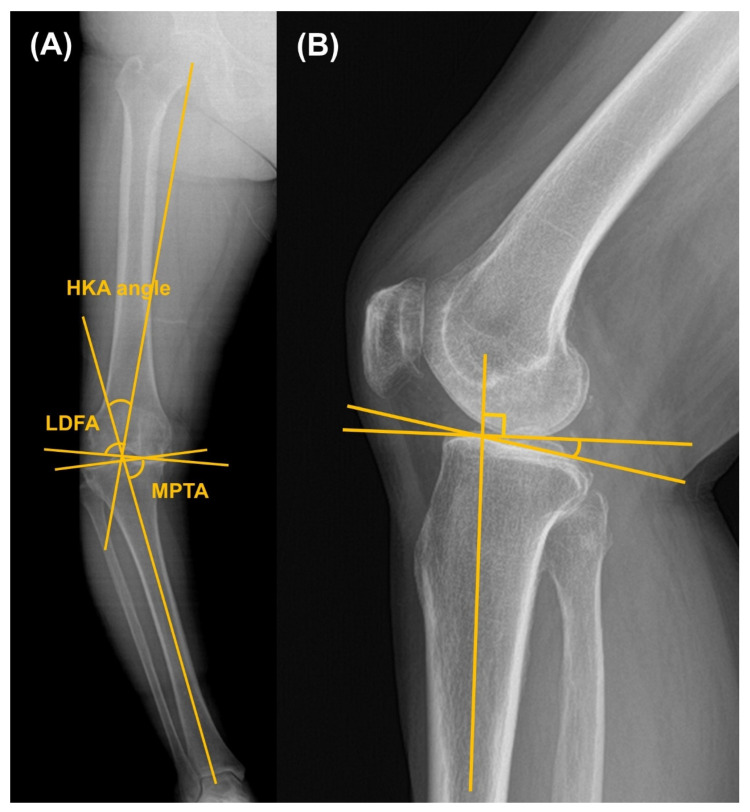
Measurement of (**A**) hip-knee ankle (HKA) angle, lateral distal femoral angle (LDFA) and medial proximal tibial angle (MPTA) on whole-leg standing radiographs and (**B**) tibial slope on knee lateral view.

**Figure 3:**
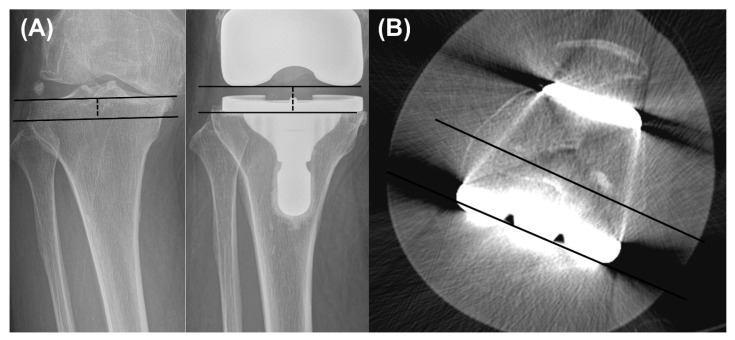
Measurement of (**A**) joint line distance on anteroposterior radiographs and (**B**) femoral component rotation angle on CT scan axial view.

Radiographic assessment was conducted using standing, full-length lower extremity radiographs acquired preoperatively and at the final follow-up. The hip–knee–ankle (HKA) angle was defined as the angle between the mechanical axis of the femur—drawn from the femoral head to the center of the knee—and the mechanical axis of the tibia—drawn from the center of the knee to the center of the talus. Positive HKA values indicated varus alignment, whereas negative values indicated valgus alignment [16]. Coronal plane morphology and implant alignment were evaluated using the lateral distal femoral angle (LDFA) and medial proximal tibial angle (MPTA) [17,18]. LDFA was measured as the angle between a line parallel to the distal femoral condyles and the femoral mechanical axis, while MPTA represented the angle between a line parallel to the tibial plateau or tibial component and the tibial anatomical axis (Figure 2). Tibial slope was determined on lateral radiographs as the angle between the mid-diaphyseal axis of the tibia and the posterior inclination of the native or implanted tibial plateau. Joint line level was assessed by measuring the perpendicular distance from the fibular head to the tibial plateau on preoperative images and to the femoral component on postoperative images [18] (Figure 3A).

Postoperative computed tomography (CT) was routinely obtained to evaluate the rotational alignment of the femoral component. The femoral component rotation angle (FCRA) was defined as the angle between the surgical transepicondylar axis (sTEA) and the line connecting the most prominent points of the medial and lateral posterior femoral condyles (Figure 3B). A higher FCRA value indicated greater external rotation, whereas a lower value reflected internal rotation. All radiographic measurements were performed using a picture archiving and communication system (Centricity; GE Healthcare, Chicago, IL, USA). Two independent orthopaedic surgeons conducted the assessments to determine interobserver reliability, and the same observers repeated all measurements after a 6-week interval to evaluate intraobserver reliability. Intraclass correlation coefficients (ICCs) demonstrated excellent consistency in both inter- and intraobserver analyses (ICC > 0.80). Radiographic outcomes, together with preoperative and postoperative clinical results, were compared between the two groups.

### 2.4. Statistical Analysis

Normality of data distribution was examined using the Shapiro–Wilk test. Paired *t*-tests were used to compare preoperative and final follow-up continuous variables, whereas categorical variables were analyzed using chi-square tests. Between-group comparisons of demographic characteristics and clinical or radiographic outcomes were performed using either Student’s *t*-test or chi-square test, as appropriate. Statistical analyses were conducted with SPSS software (version 27.0; IBM, Armonk, NY, USA), and significance was defined as *p* < 0.05. A total of 67 TKAs were included in each group. A 10-point difference in the Knee Society Score has been reported as the minimal clinically important threshold [19]. With an assumed standard deviation of 20 points, the present study achieved approximately 81% power to detect a between-group difference of at least 10 points (α = 0.05).

## 3. Results

**Figure 4:**
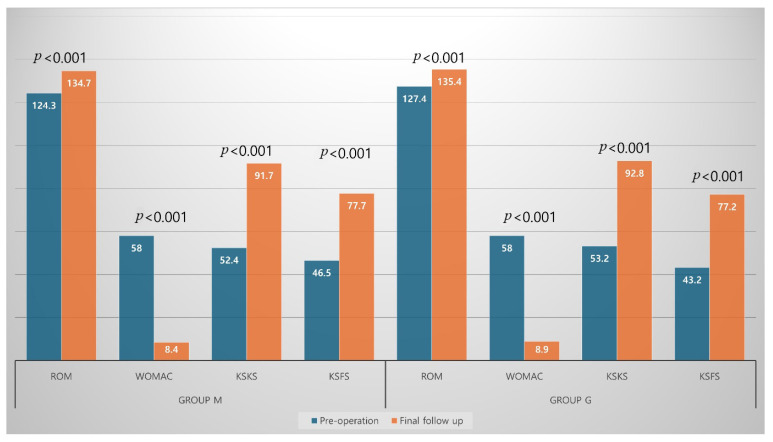
Comparison of clinical outcomes including range of motion (ROM), Western Ontario and McMaster Universities Osteoarthritis (WOMAC) index, the Knee Society Knee Score (KSKS), and the Knee Society Function Score (KSFS) between pre- and post-operation in both groups.

**Figure 5:**
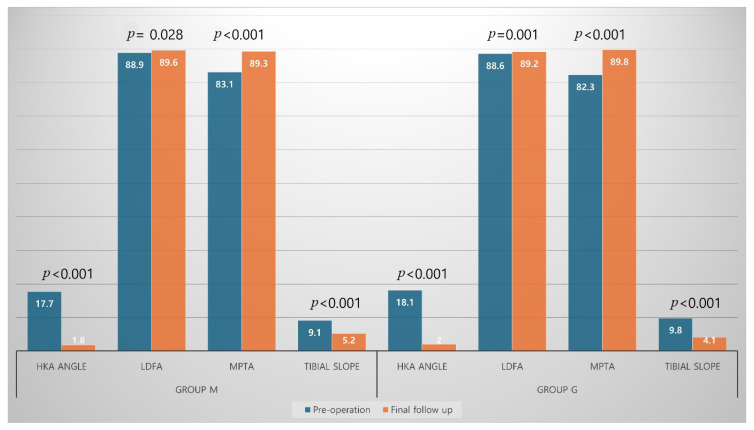
Comparison of radiologic outcomes including hip-knee-ankle (HKA) angle, lateral distal femoral angle (LDFA), medial proximal tibial angle (MPTA), and tibial slope between pre- and post-operation in both groups.

Postoperative WOMAC index, KSKS, and KSFS significantly improved compared with preoperative values in both groups (Figure 4). The postoperative HKA angle was significantly corrected, although residual varus remained in both groups. Overall, the femoral and tibial components were aligned with the mechanical axis (Figure 5). Joint line distance did not change significantly after surgery in either group (Group M: 17.6 ± 3.2 mm vs. 18.1 ± 3.8 mm, *p* = 0.508; Group G: 17.7 ± 3.5 mm vs. 18.0 ± 3.9 mm, *p* = 0.735).

Before propensity score matching, sex ratio and preoperative HKA angle were significantly different between the two groups. After matching, there were no significant differences in age, sex, BMI, or preoperative HKA angle. The mean follow-up duration was 76.2 months in Group M and 72.0 months in Group G (*p* = 0.029). Preoperative ROM, WOMAC index, KSKS, KSFS, LDFA, MPTA, tibial slope, and joint line distance did not differ significantly between the two groups (Table 1).

With respect to postoperative clinical outcomes, ROM, WOMAC index, KSKS, and KSFS were comparable between groups. Radiographically, postoperative HKA angle, LDFA, and MPTA did not differ significantly. Tibial slope was significantly greater in Group M than in Group G (5.2° ± 2.3° vs. 4.1° ± 2.3°, *p* = 0.006, Table 2). Postoperative joint line distance was similar between groups (0.11 ± 0.16 mm vs. 0.10 ± 0.17 mm, *p* = 0.415). FCRA was smaller in Group M, but the difference was not statistically significant (1.6° ± 2.1° vs. 2.1° ± 2.0°, *p* = 0.134).

No patient required revision for implant-related mechanical problems. Two patients in Group M developed periprosthetic joint infection, both of which resolved following debridement and antibiotic therapy.

## 4. Discussion

The most important finding of this study was that postoperative mid-term clinical outcomes, including ROM, WOMAC, KSKS, and KSFS, were comparable between the two groups at mid-term follow-up. Radiographic outcomes were also similar, with the exception of tibial slope.

Rectangular soft-tissue balance has long been recognized as an important factor in achieving successful mechanically aligned TKA [20]. Consequently, medial soft-tissue release is often required to obtain appropriate balance in varus knees [21]. In most cases, osteophyte removal and deep MCL release are sufficient for implantation; however, in patients with severe preoperative varus deformity, extensive medial release may be required to achieve a rectangular gap [22]. Excessive medial release has been associated with mid-flexion instability and joint line elevation, both of which are linked to postoperative knee pain and suboptimal functional outcomes [23]. A cadaveric study demonstrated that a joint line elevation of >2 mm was associated with reduced flexion angle [14], while clinical studies have shown that joint line elevation leads to patella baja and inferior clinical outcomes [23,24]. In normal knee kinematics, the lateral soft tissues are more compliant than the medial structures [15]. Sekiya et al. reported that residual lateral laxity observed in varus knees was spontaneously corrected after neutral alignment TKA [25]. For this reason, MST was introduced, with the goal of minimizing medial release while allowing residual lateral laxity. This technique has been shown to achieve excellent outcomes without joint line elevation or subjective instability [14,16]. In the present study, both groups demonstrated slight joint line elevation, but the change was not statistically significant.

TKA surgical techniques are highly standardized, with MRT and GBT both widely used to achieve satisfactory outcomes. Each method has inherent advantages and disadvantages [26]. GBT has been associated with joint line elevation and internally rotated femoral component placement, which may contribute to patellar maltracking [7,27]. These disadvantages are largely related to the need for medial release to achieve rectangular balance. We believe that preserving medial soft tissue, even in severe varus deformity, may prevent joint line elevation and excessive femoral internal rotation.

Prior cadaveric studies have shown that femoral component malrotation significantly alters knee kinematics and contact pressures [28]. Internal rotation increases medial compartment loading, while external rotation shifts loading laterally. Malrotation also reduces patellofemoral contact area and increases peak contact pressures, which may contribute to postoperative pain and impaired function. Early reports suggested that malrotation increased anterior knee pain, medial contact forces, polyethylene wear, and arthrofibrosis, leading to inferior outcomes and higher revision rates [29,30,31,32]. However, Becker et al. found no significant correlation between femoral component rotation and clinical outcomes measured by KSS and SF-36 [33]. A key distinction between MRT and GBT is the determination of femoral component rotation. In our series, both groups demonstrated slight external rotation. MST appears to have minimized the risk of internal rotation in GBT. During MRT, we referenced the transepicondylar axis, Whiteside’s line, and posterior condylar axis, but still observed a tendency toward external rotation, which may reflect surgical experience with severe varus knees and could facilitate gap balancing. While excessive malrotation is undesirable, we consider a deviation of 1.5–2° of external rotation unlikely to significantly affect clinical outcomes. In our study, a modest degree of femoral component external rotation was identified in both groups, likely reflecting surgeon-specific adjustments made to achieve optimal gap balancing. However, whether this degree of external rotation is advantageous in total knee arthroplasty for severe varus deformity remains uncertain. Further investigation is warranted.

Tibial slope is another important consideration in achieving appropriate ROM and flexion stability [34]. Tibial slope has been correlated with flexion gap, stability, and knee ROM in biomechanical studies [35]. Although an increased slope may improve flexion angle, the clinical relevance remains controversial. There is general agreement that postoperative tibial slope is not associated with patient-reported outcomes [36,37]. The surgical technique for tibial resection was identical in both groups. The observed difference in tibial slope between groups was unintentional. In this study, tibial slope was significantly greater in Group M (mean difference, 1.1°), but clinical outcomes, including ROM, WOMAC, KSKS, and KSFS, did not differ significantly between groups. This finding was not unexpected, as the between-group difference was not large and consistent with prior reports.

Before propensity score matching, the MRT group comprised 169 cases and the GBT group 67 cases. This discrepancy is attributed primarily to surgeon preference. As both surgical techniques had performed several hundred times, learning-curve issue was not expected in either group. Nonetheless, a direct comparison would have introduced imbalance in baseline characteristics, particularly the preoperative sex distribution and HKA angle. Therefore, propensity score matching was conducted to enable a more rigorous comparison between the two techniques. We believe this approach enhances the reliability of our findings.

Different implant systems were used for GBT and MRT. GBT requires more specialized instruments such as tensioners. The Physica system supplied these instruments, so this system was used for the GBT group. However, using a single implant system for both groups would have eliminated implant-related bias entirely. Both the Physica and Lospa systems utilized PS designs, and their clinical performance has been well established in prior studies. Thus, we think using different implant systems did not produce a major bias in the interpretation of our results.

Several limitations should be acknowledged. First, as mentioned above, two different implant systems were used between the groups, which may reduce the validity of the comparative outcomes. However, all procedures were performed by a single surgeon, and both implants have shown excellent outcomes in previous studies. Moreover, the primary focus of this study was alignment and component positioning rather than implant-specific outcomes. Second, the retrospective design introduces inherent biases. Third, patellar resurfacing was not routinely performed, limiting the generalizability of our findings to cases with patellar resurfacing. Fourth, our study is not a large-volume cohort and may therefore be subject to limitations in statistical power. A preoperative power analysis based on a 10-point difference in the KSS (minimal clinically important difference) demonstrated that we achieved more than 80% power. Considering the relatively low incidence of TKA performed for severe varus deformity, the present study can be regarded as a well-powered investigation of acceptable methodological quality. Fifth, both groups were evaluated at mid-term follow-up. Although the difference was statistically significant (Group M: 76.2 months; Group G: 72.0 months; *p* = 0.029), the approximately 4-month disparity is unlikely to meaningfully influence clinical outcomes such as the WOMAC index, KSKS, or KSFS. However, this difference may affect implant survival analysis and should therefore be considered when interpreting our findings. Longer-term follow-up will be necessary to more precisely determine implant survivorship. Finally, the mid-term follow-up precludes conclusions regarding implant survivorship or long-term outcomes.

## 5. Conclusions

In patients with severe varus deformity undergoing TKA with MST, clinical and radiographic outcomes, including limb alignment, joint line position, and femoral component rotation, were comparable between MRT and GBT on mid-term follow-up data. In MST TKA for severely varus knees, neither the MRT nor the GBT demonstrated clear superiority. Accordingly, even in cases of preoperative severe varus deformity, the choice of technique may be appropriately guided by the surgeon’s preference.

## Figures and Tables

**Table 1:** Comparison of demographic and pre-operative data between both groups. (before and after propensity score matching).

	Group M	Group G	*p* Value
*Before propensity score match*			
Number of patients	169	67	
Age, year	70.6 ± 6.6	71.9 ± 6.3	0.591
Sex, male:female	17:152	0:67	0.009
Body mass index, kg/m^2^	28.0 ± 3.9	27.8 ± 4.0	0.71
HKA angle, °	17.0 ± 2.8	18.1 ± 3.9	0.007
*After propensity score match*			
Number of patients	67	67	
Age, year	70.2 ± 6.0	71.1 ± 6.3	0.346
Sex, male:female	0:67	0:67	1
Body mass index, kg/m^2^	27.6 ± 3.9	27.8 ± 4.0	0.702
Follow up period, months	76.2 ± 10.4	72.0 ± 11.3	0.029
Range of motion, °	124.3 ± 22.0	127.4 ± 25.0	0.221
WOMAC index	58.0 ± 15.8	58.0 ± 15.5	0.988
KSKS	52.4 ± 7.7	53.2 ± 7.5	0.542
KSFS	46.5 ± 12.3	43.2 ± 10.9	0.102
HKA angle, °	17.7 ± 2.9	18.1 ± 3.9	0.465
LDFA, °	88.9 ± 2.5	88.6 ± 2.4	0.477
MPTA, °	83.1 ± 2.8	82.3 ± 3.2	0.148
Tibial slope, °	9.1 ± 3.4	9.8 ± 3.2	0.176
Joint line distance, mm	17.6 ± 3.2	17.7 ± 3.5	0.909

HKA, hip-knee ankle; LDFA, lateral distal femoral angle, MPTA, medial proximal tibial angle; WOMAC, Western Ontario and McMaster Universities Osteoarthritis; KSKS, Knee Society Knee Score; KSFS, Knee Society Function Score.

**Table 2:** Comparison of post-operative data between both groups.

	Group M	Group G	*p* Value
Range of motion, °	134.7 ± 17.0	135.4 ± 19.4	0.415
WOMAC index	8.4 ± 5.1	8.9 ± 5.1	0.28
KSKS	91.7 ± 6.2	92.8 ± 6.5	0.155
KSFS	77.7 ± 16.7	77.2 ± 17.8	0.434
HKA angle, °	1.8 ± 2.6	2.0 ± 2.9	0.701
LDFA, °	89.6 ± 1.7	89.2 ± 1.3	0.501
MPTA, °	89.3 ± 1.8	89.8 ± 1.6	0.063
Tibial slope, °	5.2 ± 2.3	4.1 ± 2.3	0.006
Joint line distance, mm	18.1 ± 3.8	18.0 ± 3.9	0.802
Femoral component rotation angle, °	1.6 ± 2.1	2.1 ± 2.0	0.134

HKA, hip-knee ankle; LDFA, lateral distal femoral angle, MPTA, medial proximal tibial angle; WOMAC, Western Ontario and McMaster Universities Osteoarthritis; KSKS, Knee Society Knee Score; KSFS, Knee Society Function Score.

## Data Availability

The datasets used and/or analyzed during the current study are available from the corresponding author on reasonable request.
